# Vertebral BMSC-EVs under estrogen deficiency drive senescence-related mitochondrial dysfunction in endplate chondrocytes via *MRPL1* mRNA delivery

**DOI:** 10.1038/s12276-026-01719-x

**Published:** 2026-05-07

**Authors:** Yiming Zhong, Zhuoxin Li, Haofeng Hong, Zhenda Zhao, Longting Chen, Zihuan Yang, Dongwei Fan, Wanqiong Yuan, Da Zou, Hua Tian, Chunli Song, Weishi Li, Huijie Leng

**Affiliations:** 1 Department of Orthopedics, Peking University Third Hospital, Beijing, China; 2 Department of Orthopedics, Aerospace Center Hospital, Beijing, China; 3 Beijing Key Laboratory of Spinal Diseases, Beijing, China

**Keywords:** Senescence, Experimental models of disease

## Abstract

The growing population of postmenopausal women in an aging society has led to a heightened incidence of lumbar degenerative diseases (LDD). The degeneration of the vertebral endplate cartilage is considered the initial factor in LDD, but the effect of estrogen deficiency on this process remains unclear. Here we demonstrate that estrogen deficiency triggers senescence in vertebral bone marrow mesenchymal stem cells and leads to the release of extracellular vesicles (EVs), which further accelerate the senescence of endplate chondrocytes (EPCs). Mitochondrial ribosomal proteins translate key respiratory chain components and are negatively correlated with lifespan, as their downregulation extends lifespan across species. *MRPL1*, a mitochondrial ribosomal large subunit gene, is upregulated in EV mRNA cargo under estrogen deficiency. These EVs facilitate the delivery of *MRPL1* mRNA into EPCs, enhancing MRPL1 protein translation, which in turn induces cellular senescence and supernormal mitochondrial protein turnover. MRPL1 overexpression also impairs ATP synthase activity by interacting with its catalytic subunit ATP5B, leading to senescence-related mitochondrial dysfunction in EPCs. Doxycycline administration suppresses MRPL1 expression and mitochondrial translation in EPCs, and markedly alleviates endplate degeneration associated with estrogen deficiency in a rat model, thereby introducing a novel therapeutic strategy for the management of LDD.

## MRPL1 drives senescence in vertebral stem cells

With an aging population, the prevalence of lumbar degenerative diseases (LDD) in postmenopausal women is increasing, yet the role of estrogen deficiency in LDD is not well understood. This study reveals that estrogen deficiency accelerates the senescence of vertebral bone marrow mesenchymal stem cells, which in turn promotes endplate chondrocyte senescence through extracellular vesicles. Researchers used an ovariectomized rat model to simulate estrogen deficiency and found that bone marrow mesenchymal stem cell-derived extracellular vesicles deliver *MRPL1* mRNA to endplate chondrocytes, increasing MRPL1 protein expression and mitochondrial protein turnover, impairing ATP synthesis. Doxycycline treatment inhibited MRPL1, alleviating endplate degeneration. These findings highlight the potential of targeting MRPL1 for therapeutic intervention in LDD, suggesting that further exploration of mitochondrial translation and its regulation could offer new strategies for managing age-related diseases.

This summary was initially drafted using artificial intelligence, then revised and fact-checked by the author.

## Introduction

With the advent of an aging society, the proportion of postmenopausal women is increasing. Following menopause, there is a loss of ovarian function in females and a decline in estrogen levels, which subsequently leads to bone loss and various other changes beyond the reproductive system^1,2^. Lumbar degenerative diseases (LDD) are age-related conditions, particularly prevalent in postmenopausal women^3–5^. LDD notably affect patients’ quality of life and impose a substantial economic burden on society. However, the impact of estrogen deficiency on LDD remains inadequately understood.

The vertebral endplate is a thin bilayer structure situated between the vertebra and the intervertebral disc. It consists of a layer of hyaline cartilage over a bony layer, which play a crucial role in nutrition supply and stress transmission to the intervertebral disc^6^. Endplate degeneration represents a typical manifestation of LDD and also serves as an initiating factor for this condition^7^. Therefore, the endplate is an important target for early intervention of LDD, and the impact of estrogen deficiency on this structure necessitates elucidation.

Estrogen deficiency contributes to skeletal aging, resulting in disturbances within the bone marrow microenvironment, especially concerning bone marrow mesenchymal stem cells (BMSCs)^1,8^. Stem cells, whose exhaustion is a hallmark of aging, play a crucial role in cartilage degeneration and may promote the senescence of neighboring tissues through the secretion of extracellular mediators^9–11^. Extracellular vesicles (EVs) are nanoscale membrane-bound structures that facilitate intercellular communication by transferring nucleic acids, proteins and organelle cargo between cells^12^. EVs derived from subchondral bone have been demonstrated to promote chondrocyte injury^13,14^, but the involvement of BMSC-EVs in endplate degeneration has yet to be documented.

Mitochondria, often referred to as the powerhouse of cells, play a crucial role in determining the fate and function of skeletal cells^15,16^. Mitochondria facilitate the transfer of electrons through the respiratory chain (RC), ultimately generating ATP via the F_1_F_O_ ATP synthase subunit β (ATP5B) to sustain cellular energy supply. The RC has been demonstrated to regulate cartilage growth and osteoarthritis development^17,18^. Most RC proteins are encoded by nuclear DNA and synthesized in cytoplasmic ribosomes, with the exception of 13 proteins that are encoded by mitochondrial DNA (mtDNA) and translated by mitoribosomal proteins (MRPs)^19^. MRPs function as essential modulators of both health and lifespan across various species, exhibiting remarkable evolutionary robustness^20^. Currently, research aimed at promoting health and longevity through the intervention of MRPs primarily focuses on the small subunit of the mitoribosome, with relatively less attention given to the large subunit^21,22^. MRPL1, a component of the large subunit of the mitoribosome, also exhibits a negative correlation with longevity and may thus serve as an intervention target for age-related diseases^20^. Nonetheless, the role of MRPL1 and mitochondrial translation in endplate degeneration remains to be clarified.

The current understanding of how estrogen deficiency affects endplate degeneration, along with the roles played by BMSC-EVs and the specific mechanisms involved, remains considerably limited. The present study demonstrates that that estrogen deficiency can induce the senescence of vertebral BMSCs and promote endplate chondrocyte (EPC) senescence through the secretion of EVs. BMSC-EVs deliver *MRPL1* mRNA to EPCs under estrogen deficiency, leading to aberrantly increased MRPL1 protein expression and mitochondrial protein turnover. Furthermore, MRPL1 interacts with ATP5B, thereby impairing ATP synthesis in chondrocytes. The inhibition of MRPL1 with doxycycline alleviates endplate degeneration under estrogen deficiency, offering a novel therapeutic approach for LDD.

## Materials and methods

### Animals

A total of 100 female Sprague-Dawley rats, aged 6 months and weighing 290–330 g, were obtained from the Peking University Health Science Center. They were housed two to three per cage under a controlled 12-h light/dark cycle at 25 °C and humidity of 49–55%, with free access to food and water. The animal experiments received approval from the Animal Ethics Committee of Peking University (#A2022165). The rats were randomly assigned to the following experimental groups for histological analysis and primary cell isolation: sham, ovariectomy (OVX), OVX with doxycycline treatment (OVX + Dox), and OVX with β-nicotinamide mononucleotide (NMN) treatment (OVX + NMN), as well as an aged group (24-month-old) and a corresponding young control group (6-month-old). OVX rats underwent bilateral ovariectomy, while sham rats had a sham operation^23^. Doxycycline (MCE) was administered orally at a dose of 50 mg/kg/day in drinking water starting 7 days post-surgery, while NMN (MCE) was administered orally at a dose of 300 mg/kg/day. The rats were euthanized 9 weeks after surgery.

### Cell culture and differentiation

All cells were cultured in Dulbecco’s modified Eagle medium (Gibco) with 10% fetal bovine serum and 1% penicillin–streptomycin, under 5% CO_2_ at 37 °C. For doxycycline intervention, a concentration of 30 μg/ml was applied. The estrogen receptor (ER) inhibitor fulvestrant (MCE) was used to mimic the effects of estrogen deficiency on cells.

Primary vertebral BMSCs and EPCs were obtained from sham and OVX rats. For BMSC isolation, lumbar vertebrae were collected, and the marrow cavity was rinsed with culture medium using a sterile syringe. The rinsed liquid was resuspended in culture medium, which was changed every 3 days after initial plating. Adherent cells were collected and subcultured upon reaching 80% confluence. BMSC surface markers were identified using a flow cytometry kit (OriCell). For EPC isolation, the cartilage endplates were diced and digested with 0.2% type II collagenase (Gibco) at 37 °C for 20 h. The chondrocytes were then washed and resuspended in culture medium. Sham EPCs were used as the control group or normal group.

For chondrogenic differentiation, assays were conducted in micromass cultures by placing a 10-µl droplet of cell suspension (1.5 × 10^7^ cells/ml) at the center of each well in a 24-well plate. Micromasses were incubated for 2 h before adding chondrogenic induction culture medium (OriCell). The cell medium was changed every 3 days. On day 7, the cells were fixed and subsequently stained with chondrocyte extracellular matrix (ECM) dyes, Alcian Blue (Servicebio) and Toluidine Blue (Solarbio). Microscopic images were taken to analyze the staining intensity (NIKON).

For osteogenic differentiation, the osteogenic induction culture medium (OriCell) was used. The culture medium was replaced every 3 days. At day 21, cells were fixed and stained with Alizarin Red S (Beyotime). Microscopic images were captured to analyze the proportion of the positively stained area (mineralization area).

For adipogenic differentiation, the adipogenic induction culture medium (OriCell) was used. The stimulation medium was applied for 2 days, followed by an additional day of maintenance medium. At day 21, cells were fixed and stained with Oil Red O (Solarbio). Microscopic images were captured to analyze the proportion of the positively stained area (lipid droplet area).

### Senescence-related assays

Senescent cells were identified using a SA-β-Gal assay kit (Beyotime). The EdU assay (Beyotime) was performed to analyze cell proliferation. SA-β-Gal and EdU results were analyzed by capturing microscopic images to determine the proportion of positive cells. Cell cycle analysis was conducted via flow cytometry (CytoFLEX, Beckman) with propidium iodide staining (MultiSciences). For growth rate calculation, both the initial cell number (IN) and final cell number (FN) of each passage are used in the population doubling (PD) formula (PD = [log(FN) − log(IN)]/log_2_) and then summed (cumulative population doubling).

### Migration assays

For the transwell assay, approximately 2 × 10^4^ BMSCs were seeded in the upper chamber of a 24-well transwell system (8 μm) with serum-free medium, while the lower chambers contained complete medium. After culturing for 24 h, BMSCs on the upper membrane layer were scraped off. The migrated BMSCs in the lower layer were fixed and stained with crystal violet (Beyotime). The number of migrated cells was analyzed through microscopic imaging.

For the scratch assay, BMSCs were seeded into a 12-well plate at a density of 5 × 10^5^ cells per well and scratched using a 200-μl tip. Subsequently, microphotographs of the scratches were taken, followed by another round of imaging 24 h later. The reduction in scratch area was then analyzed.

### EV isolation, identification and manipulation

EVs were purified by ultracentrifugation^24^. BMSCs from passages 3 to 6 were seeded at a density of 2 × 10^4^ cells/cm^2^ in complete medium with EV-depleted fetal bovine serum for 48 h. Cell viability was assessed via Trypan Blue staining (Beyotime), and media from cultures with >95% viability were used for EV isolation.

The BMSC culture medium was collected and centrifuged at 300*g* for 10 min at room temperature to remove cells, followed by centrifugation at 2,000*g* for 10 min at 4 °C to eliminate dead cells. The supernatant was filtered through a 0.22-μm pore filter and then centrifuged at 10,000*g* for 30 min at 4 °C to remove debris. To prepare the conditioned medium (CM), an equal volume of fresh complete medium was added.

To pellet EVs, the supernatant was ultracentrifuged at 100,000*g* for 70 min at 4 °C (Hitachi). The supernatant was then removed, and the EV pellets were washed in phosphate-buffered saline, followed by another ultracentrifugation at the same condition. The final EV pellet was resuspended in 100 μl of phosphate-buffered saline and stored at −80 °C. The protein concentration of EVs was measured using a BCA Assay Kit (Beyotime). EVs were imaged using transmission electron microscopy (Hitachi) and analyzed for particle size and concentration via nanoparticle tracking analysis (NTA; Particle Metrix). For cellular intervention, EVs were used at a protein concentration of 10 μg/ml. To inhibit EV secretion, BMSCs were pretreated with 10 μM GW4869(MCE) for 24 h. The siRNA transfection was performed 72 h before EV isolation.

### RNA extraction and qPCR

Total RNA was extracted using TRIzol reagent (Invitrogen). The cDNA was generated with cDNA Synthesis Mix (Beyotime), and quantitative polymerase chain reaction (qPCR) was performed using SYBR Green qPCR Mix (Beyotime) on a QuantStudio 3 Real-Time PCR System (Life Technologies). The relative expression of target genes was determined by the 2^−ΔΔCt^ method after normalizing to *Actb* levels. Below are the sequences of the primers used: *Actb*: 5′-CCACCATGTACCCAGGCATTG-3′/5′-GAGCCACCAATCCACACAGAG-3′; *Mrpl1*: 5′-GAAAGTGAGCCTGAAGACGAT-3′/5′-AAACACCTTGCTTTGGATTGG-3′. Actinomycin D (ACTD, MCE) was used to inhibit transcription.

### RNA sequencing and functional enrichment analysis

Total RNA from EVs was used for library preparation and sequencing at RiboBio. In brief, RNA was fragmented to approximately 200 bp, followed by first- and second-strand cDNA synthesis, adaptor ligation and low-cycle enrichment using the NEBNext Ultra RNA Library Prep Kit for Illumina (NEB). The purified library products were evaluated using the Agilent 2200 TapeStation and Qubit 2.0 (Life Technologies), followed by sequencing (2 × 150 bp) on a HiSeq3000 (Illumina). HISAT2 was used to align the clean reads to the reference genome, utilizing default parameters. HTSeq was subsequently used to convert aligned short reads into read counts for each gene model. Differential expression was assessed by DEseq using read counts as input. The Benjamini–Hochberg multiple test correction method was enabled. Differentially expressed genes were chosen according to the criteria of fold change >2 and adjusted *P* < 0.05. All differentially expressed genes were subjected to Kyoto Encyclopedia of Genes and Genomes (KEGG) pathway enrichment analysis.

### Public RNA sequencing data analysis

The single-cell RNA transcriptome datasets, including normal (GSM4878541^25^) and LDD (GSM8079184^26^) human endplate cartilage, were obtained from the Gene Expression Omnibus (GEO) database. Quality control and analysis of the data were performed using the Scanpy workflow. For the clustering of senescent chondrocytes (SnCs), the Leiden algorithm was utilized to group the cells. Subsequently, senescence-related pathways were scored to categorize closely positioned clusters into distinct senescence stages. Pseudotime analysis was conducted using the Cellrank method.

The single-cell RNA transcriptome data of BMSCs from elderly human females and males of comparable age were obtained from the GEO database (GSE253355^27^). The Seurat workflow was used to annotate BMSCs on the basis of the provided code, followed by Reactome pathway enrichment analysis and gene set enrichment analysis (GSEA) of differentially expressed genes.

The human knee cartilage GEO-seq transcriptome data were obtained from the GEO database (GSE254844^28^). The expression patterns of *MRPL* genes were analyzed using differential gene expression profiles from weight-bearing and non-weight-bearing regions.

### Transfection and infection

Transfections were conducted with Lipofectamine 3000 (Invitrogen). The sequences of the siRNA used: si-rat-*Mrpl1*#1: CGTCAGAAGTCAACAAAGT; si-rat-*Mrpl1*#2: CCTGGACTTTACCAATCCA. Nontargeting control siRNA (RiboBio) was used as a control. The pMitotimer mitochondrial turnover reporter plasmid (RRID: 52659) was obtained from Addgene. Cells were transfected and photographed under a microscope 48 h later to analyze the red–green fluorescence ratio^29,30^. To achieve stable overexpression of MRPL1 in C28/I2 cells, lentiviruses were utilized as per the manufacturer’s instructions (OBiO Biotech). The lentivirus pLenti-CMV-MRPL1-3xFLAG-PGK-BSR-WPRE was used for overexpression, while pLenti-CMV-MCS-3xFLAG-PGK-BSR-WPRE served as the control.

### Metabolism and mitochondria-related assays

The Cell Counting Kit-8 assay was used to assess cell viability (Dojindo). ATP levels were measured with a luminescent assay kit (Beyotime). ATP synthase activity was evaluated using the Complex V activity assay kit (Elabscience). The oxygen consumption rate (OCR) was determined with an oxygen probe (Bestbio).

Mitochondrial membrane potential (Δ*Ψ*_m_) level was determined using MitoTracker Red (Δ*Ψ*_m_ dependent, Thermo Fisher Scientific) by flow cytometry. Mitotracker Green (Δ*Ψ*_m_ independent, Thermo Fisher Scientific) was used for mitochondrial mass detection by flow cytometry. The mitochondrial permeability transition pore (MPTP) assay was conducted using Calcein AM staining (Beyotime) in conjunction with CoCl_2_ treatment to quench fluorescence in the cytoplasm, followed by flow cytometry analysis.

For mitochondrial protein synthesis analysis, cells were treated with 100 μg/ml cycloheximide (MCE) for 20 min to inhibit cytoplasmic translation. After washing, OP-puro (50 μM, MCE) was added for 30 min. A click reaction staining kit (Ribobio) was employed to evaluate mitochondrial protein synthesis and total protein synthesis through fluorescence intensity measured by a microplate reader (Molecular Devices).

### Antibodies

The antibodies used in this study are as follows: rabbit anti-β-actin (20536-1-AP, Proteintech), mouse anti-P16 (sc-1661, Santa Cruz), rabbit anti-P21 (ab109199, Abcam), rabbit anti-SOX9 (ab185966, Abcam), rabbit anti-COL2 (ab307674, Abcam; 28459-1-AP, Proteintech), rabbit anti-MRPL1 (PA5-109974, Thermo Fisher Scientific; 16254-1-AP, Proteintech), rabbit anti-CD81 (ab109201, Abcam), rabbit anti-TSG101 (ab125011, Abcam), rabbit Anti-LAMIN-B1 (ab133741, Abcam), rabbit IgG control antibody (30000-0-AP, Proteintech), rabbit anti-ATP5B (ab170947, Abcam; 17247-1-AP, Proteintech), rabbit anti-GAPDH (10494-1-AP, Proteintech), horseradish peroxidase (HRP)-conjugated goat anti-rabbit IgG (SA00001-2, Proteintech; G1213, Servicebio), HRP-conjugated goat anti-mouse IgG (SA00001-1, Proteintech; G1214, Servicebio) and mouse anti-rabbit IgG (Conformation Specific) (5127S, CST).

### Western blot

Cells were lysed in RIPA buffer with protease inhibitors (Beyotime) for 30 min. After centrifugation at 10,000*g* for 20 min at 4 °C, the supernatant was collected. Protein concentration was determined using a BCA assay kit (Beyotime). Sodium dodecyl sulfate–polyacrylamide gel electrophoresis (PAGE) was performed to load and separate samples. Proteins were transferred to polyvinylidene fluoride membranes (Millipore). The membranes were blocked with 5% nonfat milk for 1 h at room temperature and then incubated overnight with primary antibodies at 4 °C. An incubation with HRP-conjugated secondary antibody was conducted for 1 h at room temperature. Subsequently, exposure was performed using an enhanced chemiluminescence kit (Beyotime).

### Co-immunoprecipitation assay

Cell lysates were precipitated with anti-Flag or protein A/G magnetic beads following the manufacturer’s instructions (Beyotime) and analyzed by western blot.

### BN-PAGE

Blue native (BN)-PAGE was conducted following a published protocol^31^. To enhance ATP synthase isolation, pelleted mitochondria were solubilized with Triton X-100 at a ratio of 3 g/g.

### Mass spectrometry

Protein samples from immunoprecipitation (IP) and BN-PAGE bands of mitochondrial ATP synthase (approximately 592 kDa) were analyzed using an Orbitrap Exploris 480 mass spectrometer (ThermoFisher Scientific). The results were further evaluated using the STRING database.

### Molecular docking and molecular dynamics simulations

The ZDock3 algorithm was used to perform a global docking of MRPL1 (PDB: 7A5K^32^) and ATP5B (PDB: 8H9E^33^), utilizing rigid docking with an angle step of 15°. The model with the highest score was selected for further analysis.

According to the molecular docking model, molecular dynamics simulations were conducted using GROMACS. The parameters were as follows: force field: Amber14sb; system box: cubic; energy minimization: 30,000 steps; canonical ensemble: 100 ps at 310 K; isobaric–isothermal ensemble: 100 ps at 1 bar; production run: simulation for 100 ns (500,000 steps × 0.002 fs per step).

The binding free energy (Δ*G*) was calculated using the GMX-MMPBSA method, analyzing the last 80–100 ns of the trajectory and computing every 20 frames.

### MicroCT analysis

Lumbar vertebrae were scanned using micro-computed tomography (microCT; Siemens) with the following parameters: 13 μm isotropic voxel, 80 kV voltage, 500 μA current, 300 ms settling time, 360 projections per 360° and 200 ms exposure time. Raw data were reconstructed using Inveon Acquisition Workplace (Siemens). The L4 caudal bony endplate was selected as the region of interest. Bone porosity was calculated with Inveon Research Workplace (Siemens). The disc height index (DHI) was calculated by dividing the intervertebral disc height by the average height of the adjacent vertebrae.

### Histomorphometry and immunohistochemistry

L4–5 vertebrae were dissected and fixed in 4% paraformaldehyde for 48 h. The tissue was decalcified in 10% EDTA (pH 7.4) for 3 weeks before being embedded in paraffin and cut into 4-µm-thick sections. After safranin O/Fast Green (S&F) staining, endplate scores were evaluated^34^. For immunostaining, paraffin sections were deparaffinized and hydrated, followed by antigen retrieval. Subsequently, the sections underwent blockage of endogenous peroxidase activity and serum blocking. The sections were then incubated overnight at 4 °C with primary antibodies, followed by a 1-h incubation at room temperature with HRP-conjugated secondary antibodies. A DAB Kit (Servicebio) was used to detect immunoreactivity, followed by hematoxylin counterstaining (Servicebio). Finally, the sections were dehydrated, coverslipped and microscopically analyzed for staining intensity or positivity rates.

### Statistical analysis

Results are presented as the mean ± standard deviation (s.d.). Statistical analyses were performed using GraphPad Prism 10 (GraphPad Software). The Student’s *t*-test was used to assess significant differences between two groups. For the analysis involving three or more groups, one-way analysis of variance with Tukey’s comparison post-hoc test was used. Results with *P* < 0.05 were used as the threshold for significance.

## Results

### EVs derived from OVX-BMSCs promote senescence in EPCs

**Fig. 1:**
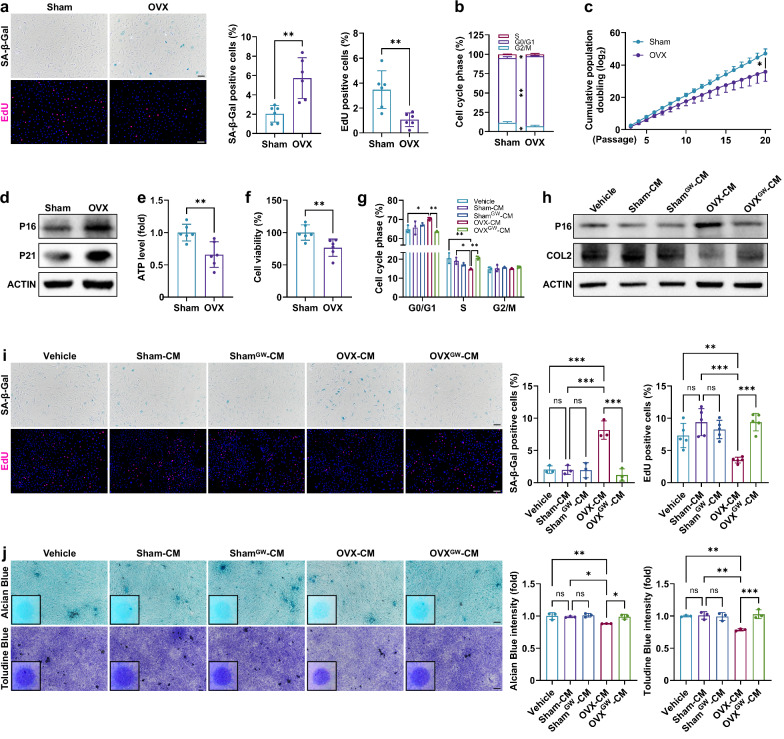
EVs from OVX-BMSCs induce EPC senescence. **a**, SA-β-Gal and EdU staining of vertebral BMSCs from sham-operated and OVX rats (*n* = 6). **b**, Cell cycle analysis of BMSCs (*n* = 3). **c**, Growth curves of BMSCs (*n* = 4). **d**, Western blot analysis of BMSCs. **e**, ATP levels of BMSCs (*n* = 6). **f**, Cell viability of BMSCs (*n* = 6). **g**, Cell cycle analysis of rat EPCs after BMSC-CM treatment for 3 days (*n* = 3). GW, GW4869. **h**, Western blot analysis of EPCs (3 days). **i** SA-β-Gal (*n* = 3) and EdU (*n* = 5) staining of EPCs (3 days). **j**, Alcian Blue and Toluidine Blue staining of EPCs cultured using the micromass method (7 days, *n* = 3). Error bars represent the mean ± s.d. *P < 0.05, **P < 0.01, ***P < 0.001; ns, not significant. Scale bar, 100 μm.

The impact of estrogen deficiency on the senescence of vertebral BMSCs was investigated using an ovariectomized (OVX) rat model. The flow cytometry results indicate that the extracted cells possess typical BMSC surface markers, being positive for CD90, CD73, CD44 and CD29, while negative for CD45, CD34 and CD11b/c (Supplementary Fig. 1a). Estrogen deficiency significantly increased senescence-associated β-galactosidase (SA-β-Gal) expression (Fig. 1a), inhibited cell proliferation (EdU; Fig. 1a), exacerbated cell cycle arrest (S-phase decline or G0/G1 increase; Fig. 1b) and reduced growth rate in OVX-BMSCs (Fig. 1c). In addition, the expression levels of senescence-associated proteins P16 and P21 were elevated (Fig. 1d), while ATP content and cell viability decreased in OVX-BMSCs (Fig. 1e,f). The results indicate that estrogen deficiency significantly accelerates BMSC senescence.

The differentiation potential of BMSCs is crucial for cartilage maintenance^35^. Compared with sham-BMSCs, OVX-BMSCs exhibited decreased chondrogenic capacity, as indicated by a reduction in the production of ECM (Supplementary Fig. 1b) and a decrease in the expression levels of the chondrogenic transcription factor (SOX9) and the ECM component COL2 (Supplementary Fig. 1e). In addition, its osteogenic differentiation ability decreases (Supplementary Fig. 1c) while adipogenic differentiation ability increases (Supplementary Fig. 1d), consistent with the characteristics of senescent BMSCs^36^. Moreover, the migration ability of OVX-BMSCs was diminished (Supplementary Fig. 1f,g). Decreased chondrogenic potential and migratory capability may contribute to the degeneration of cartilage.

To further validate the effects of estrogen deficiency, transcriptomic analysis was performed using public data to compare gene expression profiles of BMSCs in elderly women and age-matched men^27^. The Reactome enrichment analysis results, in conjunction with the GSEA findings, also demonstrated a significant downregulation of ECM-related pathways in BMSCs derived from elderly women as a consequence of estrogen deficiency (Supplementary Fig. 1h,i). This finding aligns with our observation of reduced ECM expression during OVX-BMSC differentiation. The ECM is currently recognized as an important factor in cellular senescence and has been classified as one of the new hallmarks of aging^37^. Therefore, estrogen deficiency-induced alterations in the ECM may serve as a key contributor to BMSC senescence.

To further validate the role of estrogen deficiency in endplate aging, in vivo aging and anti-aging interventions were conducted. OVX rats displayed endplate aging characteristics similar to aged rats (Supplementary Fig. 2a,c), including elevated cartilage endplate degeneration score, increased bony endplate porosity and reduced intervertebral disc height (DHI). In addition, the anti-aging compound NMN, as a precursor to NAD^+^, improved endplate aging in OVX rats. This effect may stem from NMN’s ability to restore the age-related decline in NAD^+^ levels^38^, which could be attributed to the secretome of vertebral BMSCs, based on results from BMSC-derived CM interventions on EPCs (Supplementary Fig. 2b). These findings indicate that estrogen deficiency contributes to the degenerative processes linked with endplate aging induced by vertebral BMSCs.

The effects of EVs derived from OVX-BMSCs (OVX-EVs) on EPCs were subsequently investigated. Compared with CM from sham-operated BMSCs (sham-CM), OVX-CM intensified cell cycle arrest, elevated P16 protein levels, decreased COL2 protein levels, increased SA-β-Gal expression, hindered cell proliferation and inhibited ECM production in EPCs (Fig. 1g–j). Inhibition of BMSC-EV secretion using GW4869 alleviated the adverse effects of OVX-CM on EPCs. To exclude the direct effect of estrogen deficiency on EPC senescence, we used the ER inhibitor fulvestrant to mimic estrogen deficiency in vitro^1,2,39^. The results demonstrated that, under ER inhibition, BMSCs exhibited significant senescence, whereas EPCs remained unaffected, despite both cell types experiencing similar levels of cytotoxicity (Supplementary Fig. 2d–f). Moreover, inhibition of ERs in BMSCs recapitulated their pro-aging effects on EPCs (Supplementary Fig. 2g). Therefore, EPC senescence may not be directly attributable to estrogen deficiency acting on EPCs themselves, but rather results from the paracrine effects of other senescent cells, specifically BMSCs. These findings suggest that estrogen deficiency accelerates the senescence of vertebral BMSCs, which in turn promotes EPC senescence through EV-mediated signal transduction.

### *MRPL1* mRNA is elevated in OVX-EVs and SnCs

**Fig. 2:**
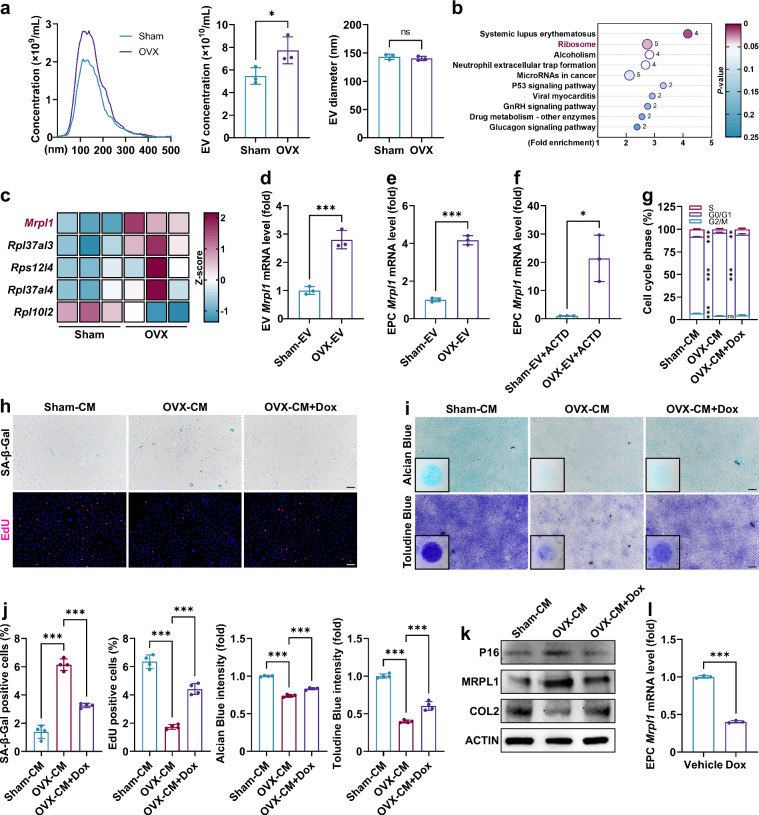
*Mrpl1* mRNA expression is significantly increased in OVX-EVs. **a**, Size distribution, concentration and diameter of isolated BMSC-EVs assessed by NTA (*n* = 3). **b**,**c**, KEGG enrichment analysis based on differentially expressed mRNAs in BMSC-EVs. **d**, *Mrpl1* mRNA expression in BMSC-EVs (*n* = 3). **e**,**f**, *Mrpl1* mRNA expression in EPCs treated with BMSC-EVs (24 h) and ACTD (pretreat EPCs with 1 μg/ml for 6 h) (*n* = 3). **g**, Cell cycle analysis of EPCs after BMSC-CM and doxycycline treatment (3 days, *n* = 6). **h**–**j**, SA-β-Gal staining (3 days), EdU staining (3 days), Alcian Blue staining (7 days) and Toluidine Blue (7 days) staining of EPCs (*n* = 4). **k**, Western blot analysis of EPCs (3 days). **l**, *Mrpl1* mRNA expression in EPCs treated with doxycycline (3 days, *n* = 3). Error bars represent mean ± s.d. *P < 0.05, **P < 0.01, ***P < 0.001; ns, not significant. Scale bar, 100 μm.

The alterations in BMSC-EVs were further examined to elucidate the underlying molecular mechanisms. EVs were identified through transmission electron microscopy and western blot (Supplementary Fig. 3a,b). NTA revealed an increase in EV secretion by OVX-BMSCs (Fig. 2a), consistent with observation in senescent cells^40^. RNA sequencing of sham-EVs and OVX-EVs showed significant enrichment of ribosome-related mRNAs, particularly *Mrpl1*, which demonstrated a remarkable upregulation of expression in OVX-EVs (Fig. 2b,c and Supplementary Fig. 3c). To identify the key contributing factor, the activities of cytoplasmic ribosomal proteins (RPs) and MRPs were assessed after EV intervention (Supplementary Fig. 3d,e). The results showed that OVX-EVs significantly enhance mitochondrial ribosome translation in EPCs without affecting total (mainly cytoplasmic) protein translation. Consequently, MRPL1 and mitochondrial ribosomes were prioritized for subsequent experiments. The qPCR results verified the high expression of *Mrpl1* mRNA in OVX-EVs (Fig. 2d). The intervention with OVX-EVs significantly increased the levels of *Mrpl1* mRNA in EPCs, regardless of whether transcription was suppressed, thereby demonstrating that this increase is attributed to the delivery of nucleic acids from EVs (Fig. 2e,f). In the presence of the mitoribosome inhibitor doxycycline, the pro-aging effect of OVX-CM on EPCs was alleviated, and both MRPL1 protein and *Mrpl1* mRNA expression in EPCs were suppressed (Fig. 2g–l). Therefore, EV-*MRPL1* might play a key role in promoting EPC senescence.

**Fig. 3:**
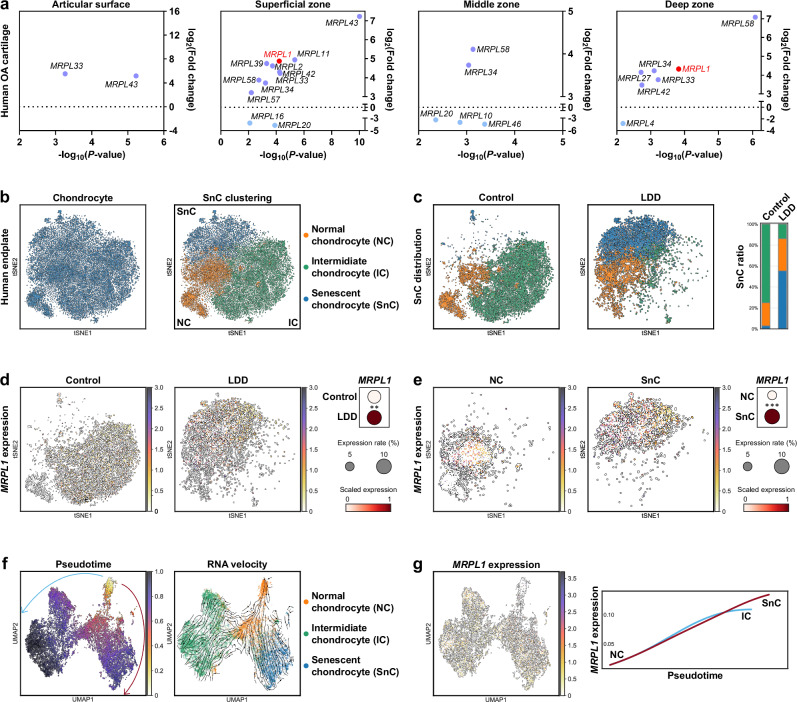
Single-cell analysis of *MRPL1* mRNA expression in cartilage degeneration and aging. **a**, GEO-seq transcriptome analysis of human knee cartilage. **b**, Clustering of SnCs in human endplate cartilage through single-cell transcriptomic analysis. **c**, Proportion of senescent EPCs. **d**,**e**, Expression of *MRPL1* mRNA in EPCs. **f**, Pseudotime analysis of the EPC senescence process. Arrows indicate the differentiation direction. **g**, Pseudotime analysis of *MRPL1* mRNA levels in EPCs. **P < 0.01, ***P < 0.001.

The expression levels of *MRPL1* mRNA were further examined in clinical samples^28^. We compared the publicly available transcriptomic profiles of osteoarthritic knee cartilage from different layers, focusing on degenerated (weight-bearing) versus nondegenerated (non-weight-bearing) regions (Fig. 3a). The results showed that, in degenerated cartilage, the mRNA expression of the mitochondrial ribosomal large subunit was generally upregulated. In particular, in the superficial and deep layers of cartilage—regions where EVs can readily access the tissue via synovial fluid and subchondral bone vasculature—the mRNA expression of *MRPL1* was significantly upregulated, further supporting its transcellular origin.

Single-cell analysis was used to clarify the association between *MRPL1* expression and endplate degeneration^25,26^. We integrated normal and LDD human EPCs from public data, clustering them into three groups by senescence level: normal chondrocytes (NCs), intermediate chondrocytes (ICs) and SnCs (Fig. 3b and Supplementary Fig. 4a–c). *MRPL1* mRNA expression is significantly elevated in LDD EPCs compared with normal EPCs (Fig. 3d). The senescence of LDD EPCs is markedly increased, and the expression of *MRPL1* is also notably upregulated in SnCs (Fig. 3c,e). Moreover, pseudotime analysis revealed a progressive transition from NCs to ICs over time, with a subset of these ICs further differentiating into SnCs (Fig. 3f). The expression of *MRPL1* exhibited corresponding changes during this period. As EPCs transitioned from NC to IC, the expression level of *MRPL1* gradually increased. In ICs, the maintenance of the IC state is accompanied by a gradual stabilization in the expression of *MRPL1*. Conversely, in cells undergoing further transition to SnCs, the expression of *MRPL1* exhibits additional increases (Fig. 3g). Therefore, the upregulation of *MRPL1* mRNA may have profound implications for EPC senescence and act as its initiating factor.

### Knockdown of MRPL1 attenuates senescence in EPCs

**Fig. 4:**
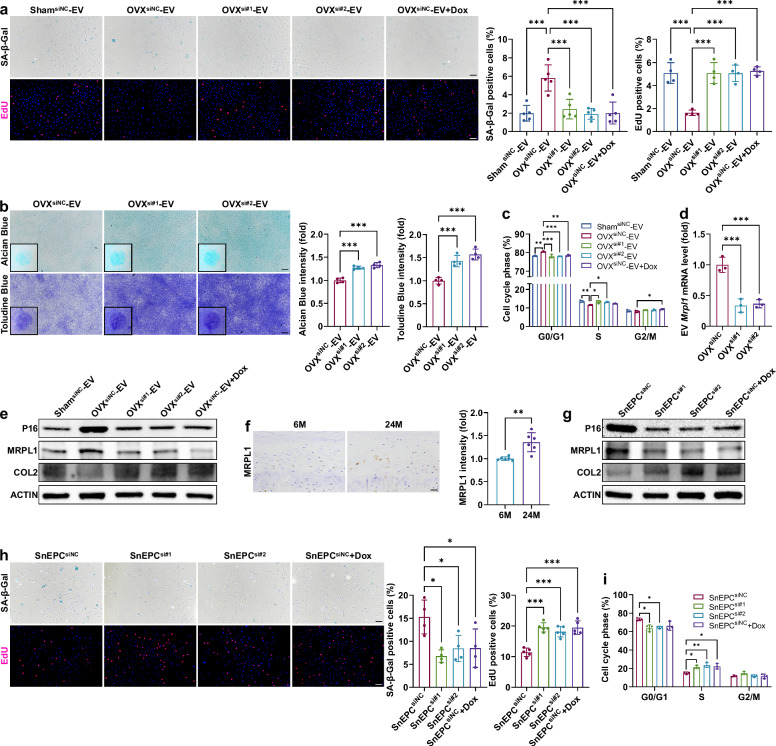
MRPL1 knockdown reduces EPC senescence. **a**, SA-β-Gal (*n* = 5) and EdU (*n* = 4) staining of EPCs after BMSC-EV and doxycycline treatment (3 days). Scale bar, 100 μm. **b**, Alcian Blue and Toluidine Blue staining of EPCs (7 days, *n* = 4). Scale bar, 100 μm. **c**, Cell cycle analysis of EPCs (3 days, *n* = 4). **d**, *Mrpl1* mRNA levels in EVs from *Mrpl1*-siRNA-treated BMSCs (1 day, *n* = 3). **e**, Western blot analysis of EPCs (3 days). **f**, Immunohistochemical analysis of endplates from young (6 months) and aged (24 months) rats (*n* = 6). M, month. Scale bar, 25 μm. **g**, Western blot analysis of *Mrpl1*-siRNA and doxycycline-treated senescent EPCs (SnEPCs) (3 days). **h**, SA-β-Gal (*n* = 4) and EdU (*n* = 5) staining of SnEPCs (3 days). Scale bar, 100 μm. **i**, Cell cycle analysis of SnEPCs (*n* = 3). Error bars represent mean ± s.d. *P < 0.05, **P < 0.01, ***P < 0.001.

To determine the role of EV-*MRPL1* in EPC senescence, siRNA-mediated knockdown was used to reduce *Mrpl1* mRNA expression in OVX-BMSCs, thereby inhibiting EV-*Mrpl1* (Fig. 4d). This knockdown significantly reduced MRPL1 protein expression in EPCs and alleviated senescence-related phenotypes after EV treatment (Fig. 4a–c,e). The incorporation of doxycycline intervention with EPCs similarly suppressed the pro-aging effects of OVX-EVs on EPCs and reduced MRPL1 protein expression in these cells. In addition, immunohistochemistry results revealed that EPCs from aged rats exhibit higher MRPL1 expression levels compared with those from young rats (Fig. 4f). To further elucidate its role in the aging process, we knocked down MRPL1 in senescent EPCs (passage 8) and applied doxycycline treatment. Both interventions significantly reduced MRPL1 expression and senescence-related phenotypes in senescent EPCs (Fig. 4g–i). Therefore, MRPL1 may serve as a valuable target for aging interventions in EPCs.

### Overexpression of MRPL1 promotes chondrocyte senescence and mitochondrial protein turnover

**Fig. 5:**
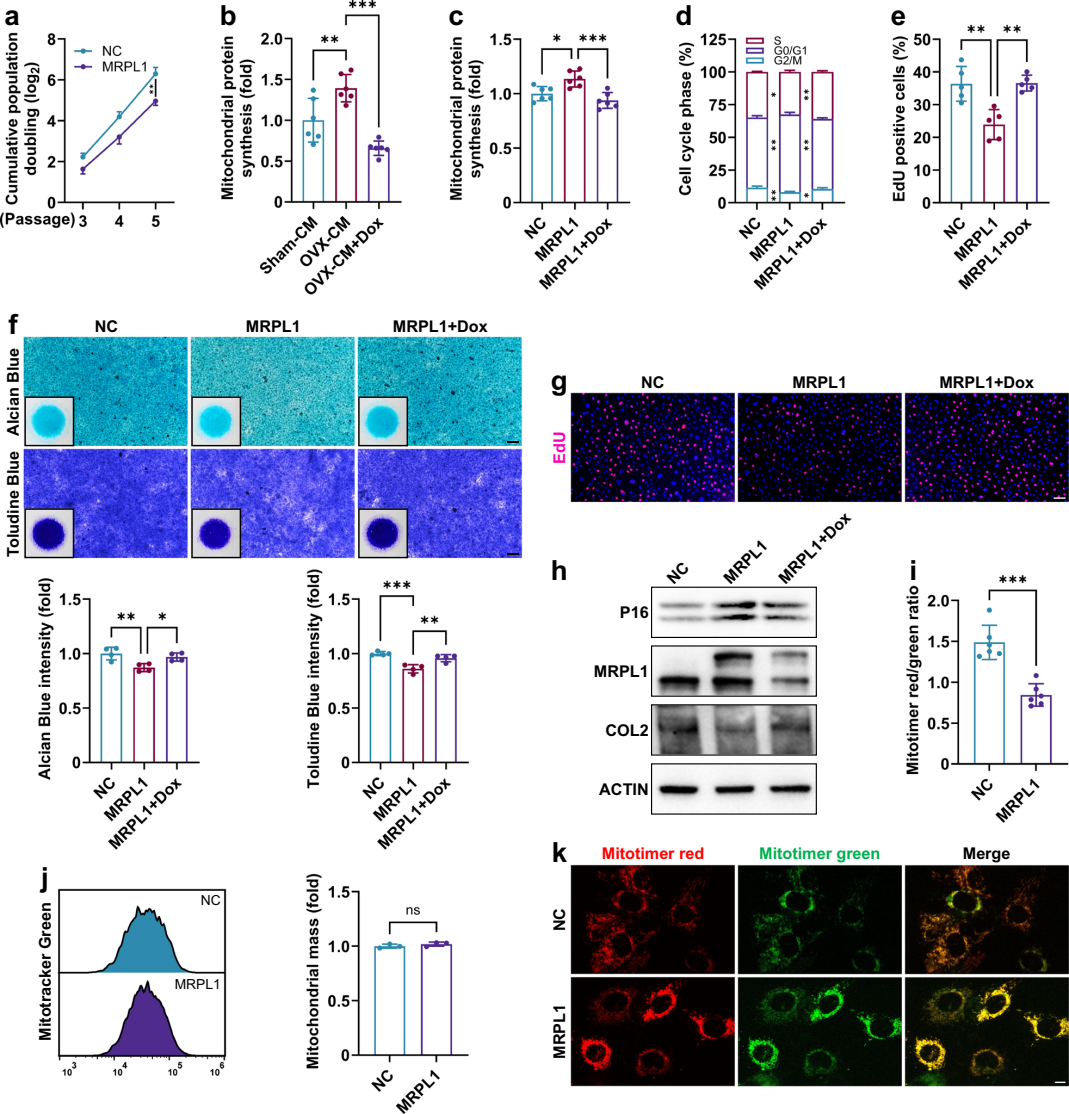
MRPL1 overexpression accelerates chondrocyte senescence and mitochondrial protein turnover. **a**, Growth curves of C28/I2 human chondrocytes after MRPL1 overexpression (*n* = 3). **b**, Mitochondrial protein synthesis of EPCs after BMSC-CM and doxycycline treatment (3 days, *n* = 6). **c**, Mitochondrial protein synthesis of MRPL1-overexpressed C28/I2 cells after doxycycline treatment (3 days, *n* = 6). **d**, Cell cycle analysis of C28/I2 cells (3 days, *n* = 3). **e**,**g**, EdU staining of C28/I2 cells (3 days, *n* = 5). Scale bar, 100 μm. **f**, Alcian Blue and Toluidine Blue staining of C28/I2 cells (3 days, *n* = 4). Scale bar, 100 μm. **h**, Western blot analysis of C28/I2 cells (3 days). **i**,**k**, Mitotimer analysis for mitochondrial protein turnover of C28/I2 cells (2 days, *n* = 6). Scale bar, 12.5 μm. **j**, Mitochondrial mass analysis of C28/I2 cells (3 days, *n* = 3). Error bars represent mean ± s.d. *P < 0.05, **P < 0.01, ***P < 0.001; ns, not significant.

To observe the direct effects of MRPL1, it was overexpressed in C28/I2 human chondrocytes via lentivirus, and senescence-related phenotypes were recapitulated (Fig. 5a,d–h). Doxycycline intervention alleviated chondrocyte senescence (Fig. 5d–h). Overexpression of MRPL1 enhanced mitoribosome function and increased mitochondrial synthesis, and this effect was inhibited by doxycycline (Fig. 5c). Similar results were observed in OVX-CM-treated EPCs (Fig. 5b). Although mitochondrial protein translation was increased, there was no significant change in mitochondrial mass (Fig. 5j). Thus, mitochondrial protein turnover may have been modulated to preserve mitochondrial mass. The results from Mitotimer confirmed that the overexpression of MRPL1 diminished the ratio of aged (red) to newly synthesized (green) mitochondrial proteins (Fig. 5i,k), suggesting an accelerated turnover of mitochondrial proteins^29,30^, which may potentially lead to mitochondrial dysfunction.

### Overexpression of MRPL1 impairs ATP synthase activity through its interaction with ATP5B

**Fig. 6:**
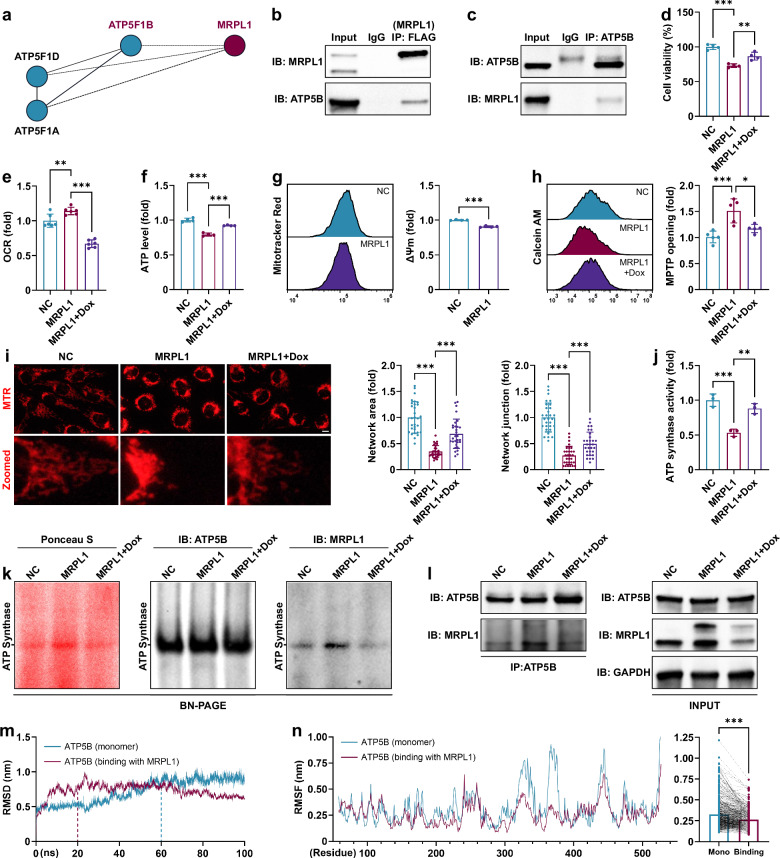
MRPL1 overexpression impairs ATP synthase activity by interacting with ATP5B. **a**, STRING analysis of MRPL1 and mitochondrial ATP synthase proteins detected by MS after FLAG (MRPL1) IP of MRPL1-overexpressed C28/I2 cells. **b**, Co-IP analysis of MRPL1-overexpressed C28/I2 cells. **c**, Co-IP analysis of control C28/I2 cells. **d**, Cell viability of MRPL1-overexpressed C28/I2 cells after doxycycline treatment (3 days, *n* = 4). **e**, OCR of C28/I2 cells (3 days, *n* = 6). **f**, ATP levels of C28/I2 cells (3 days, *n* = 4). **g**, Mitochondrial membrane potential (Δ*Ψ*_m_) of C28/I2 cells (*n* = 4). **h**, MPTP opening of C28/I2 cells (3 days, *n* = 5). **i**, Mitochondrial dynamics of C28/I2 cells (3 days, *n* = 30). MTR, MitoTracker Red. **j**, ATP synthase activity of C28/I2 cells (3 days, *n* = 3). **k**, BN-PAGE isolation of ATP synthases from C28/I2 cells and subsequent western blot analysis (3 days). **l**, Co-IP analysis of C28/I2 cells (3 days). **m**,**n**, Molecular dynamics simulation of ATP5B. Error bars represent mean ± s.d. *P < 0.05, **P < 0.01, ***P < 0.001. Scale bar, 12.5 μm.

To further elucidate the mechanism by which MRPL1 induces mitochondrial dysfunction, we performed IP to pull down MRPL1 and conducted mass spectrometry (MS) analysis to identify interacting proteins. The results suggested that MRPL1 interacts with ATP5B, the catalytic subunit of F_1_F_O_ ATP synthase (Fig. 6a). Co-IP and molecular docking (Δ*G* = −67.95 kJ/mol) further confirmed that this interaction is robust (Fig. 6b,c and Supplementary Fig. 5a,b). Next, the metabolic effects of MRPL1 were examined. MRPL1 overexpression decreased cell viability (Fig. 6d) and increased oxygen consumption (Fig. 6e) but inhibited ATP production (Fig. 6f) and mitochondrial membrane potential (Δ*Ψ*_m_) (Fig. 6g), indicating ATP synthase dysfunction. Doxycycline, by contrast, enhanced cell viability, inhibited oxygen consumption and promoted ATP production. Similar results were observed during the culture of EPCs with OVX-CM (Supplementary Fig. 5e–h). Further examination of ATP synthase activity revealed that overexpression of MRPL1 inhibited ATP synthase activity, which was alleviated by doxycycline (Fig. 6j).

ATP synthases regulate MPTP and mitochondrial dynamics^41,42^, substantially influencing mitochondrial protein turnover. Consistent with our hypothesis, our results indicate that MRPL1 overexpression promoted MPTP opening (Fig. 6h) and disrupted the mitochondrial network (Fig. 6i), while doxycycline intervention improved these phenomena. Moreover, through BN-PAGE and MS, we observed the binding of MRPL1 to ATP synthases (Fig. 6k and Supplementary Fig. 5c,d). In addition, with the incorporation of co-IP results, we found that the overexpression of MRPL1 enhanced its binding to ATP synthases and ATP5B, which was diminished following treatment with doxycycline (Fig. 6k,l). The results imply that the enhanced binding of MRPL1 to ATP5B disrupts ATP synthase function, leading to a series of mitochondrial dysfunctions associated with senescence.

We further used molecular dynamics simulations to validate the aforementioned conclusions. The stability of the MRPL1–ATP5B complex was first analyzed. The results indicated that its radius of gyration (indicative of compactness), solvent-accessible surface area (reflecting the area exposed to solvent) and number of hydrogen bonds (suggestive of interaction stability) all reached a relative equilibrium state during the simulation period, suggesting that the complex exhibits considerable stability (Supplementary Fig. 5i–k). Following that, the impact of MRPL1 on the flexibility of ATP5B was investigated. The results demonstrated that the interaction with MRPL1 notably reduced the time required for ATP5B to reach a stable state, as indicated by its root mean square deviation (RMSD), suggesting an increase in protein rigidity and a decline in flexibility (Fig. 6m). Furthermore, the root mean square fluctuation (RMSF) of ATP5B was evaluated to assess the flexibility of each amino acid residue within the protein. The results revealed that MRPL1’s interaction markedly diminished the overall flexibility of ATP5B’s amino acid residues, thereby reinforcing the notion that protein flexibility is compromised (Fig. 6n). During ATP synthesis, ATP5B exhibits high conformational variability^33^. Thus, the reduction in ATP5B’s flexibility induced by the interaction with MRPL1 is likely to impede the functionality of ATP synthases, ultimately leading to corresponding senescence-related mitochondrial dysfunction.

### Doxycycline mitigates endplate degeneration under estrogen deficiency

**Fig. 7:**
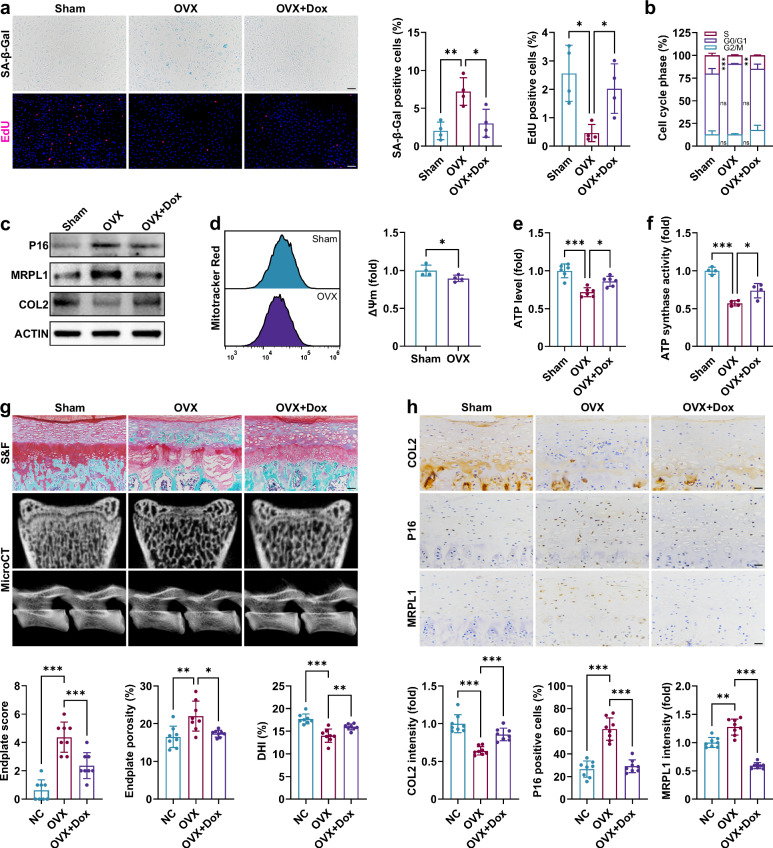
Doxycycline alleviates endplate degeneration under estrogen deficiency. **a**, SA-β-Gal and EdU staining of EPCs from OVX rats and treated with doxycycline (3 days, *n* = 4). Scale bar, 100 μm **b**, Cell cycle analysis of EPCs (3 days, *n* = 3). **c**, Western blot analysis of EPCs (3 days). **d**, Mitochondrial membrane potential (Δ*Ψ*_m_) of EPCs (3 days, *n* = 4). **e**, ATP levels of EPCs (3 days, *n* = 6). **f**, ATP synthase activity of EPCs (3 days, *n* = 4). **g**, S&F staining and microCT analysis of rat endplates (*n* = 8). Scale bar, 50 μm. **h**, Immunohistochemistry analysis of rat endplates (*n* = 8). Scale bar, 25 μm. Error bars represent mean ± s.d. *P < 0.05, **P < 0.01, ***P < 0.001; ns, not significant.

Our subsequent investigation validated the pharmacological effects of doxycycline. Doxycycline intervention was administered to primary cells following the establishment of the OVX model. In OVX-BMSCs, doxycycline promoted chondrogenic differentiation without significantly affecting their migration ability (Supplementary Fig. 6a,b,c,e). In OVX-EPCs, doxycycline alleviated senescence-related phenotypes and inhibited MRPL1 expression (Fig. 7a–c). Estrogen deficiency leads to a decline in ATP synthase activity in EPCs, which can be reversed by doxycycline (Fig. 7d–f). Furthermore, in vivo experiments demonstrated that doxycycline significantly slowed down endplate degeneration caused by estrogen deficiency, suppressed MRPL1 and P16 expression, and upregulated COL2 expression (Fig. 7g,h), thereby exhibiting promising therapeutic effects without evident toxicity (Supplementary Fig. 6d).

## Discussion

This study identifies that EVs derived from senescent vertebral BMSCs act as a pro-aging factor that promotes the senescence of EPCs under estrogen deficiency. Senescence, defined as a cellular state marked by irreversible cell cycle arrest, is frequently observed in the context of age-related diseases. Senescence resulted in elevated levels of SA-β-Gal, decreased cell proliferation and increased expression of P16^43^. Senescent BMSCs play an important role in age-related degeneration of mesoderm-derived bone and joint tissues, which is an important therapeutic target for rejuvenation^10^. MSC-EVs have been widely endorsed as an effective therapeutic approach for tissue regeneration and repair^44^. However, EVs can also function as pro-aging mediators secreted by senescent cells into the surrounding microenvironment, a phenomenon known as the senescence-associated secretory phenotype^45^. The functional impacts of MSC-EVs have been reported to be contingent upon the delivery of their mRNA cargo^46,47^, although limited information is available regarding whether and how BMSC-EVs influence EPCs. The nutritional supply to EPCs is primarily derived from the subchondral bone microenvironment, while BMSCs play a vital role in the bone marrow niche and are sensitive to estrogen^1,6,48^. Thus, changes in BMSC-EVs should have a substantial impact on EPCs, as validated by this study showing that the modification of the mRNA cargo of BMSC-EVs under estrogen deficiency effectively induces EPC senescence.

The results show that vertebral BMSC-derived EVs under estrogen deficiency contain higher levels of *MRPL1* mRNA, which enhances MRPL1 expression in EPCs and triggers senescence. The mitochondrion is an essential organelle for numerous pivotal cellular processes such as metabolism and ATP synthesis, which occur through reactions facilitated by RC protein complexes partially encoded by mtDNA^19^. The synthesis of proteins encoded by mtDNA relies on MRPs, which assemble to form mitoribosomes^49^. According to the endosymbiotic theory, human mitoribosomes share similarities with bacterial ribosomes, and their translation activity can be inhibited by antibiotics such as doxycycline^50,51^. MRPs have been identified as key regulators of metabolism and longevity. Antibiotics that target mitochondrial translation pharmacologically mimic the effects of MRP knockdown, thereby extending lifespan across various species^20^. This study identified a potential correlation between increased *MRPL1* mRNA and endplate degeneration as well as aging through single-cell and GEO-seq analysis. In addition, by regulating EV-*MRPL1* expression, its pro-aging effects on EPCs were demonstrated. There is currently no existing research concerning the transcellular regulation of MRPs in aging tissues, and our findings may offer new insights into their role in endplate degeneration and other age-related diseases.

MRPL1, as a novel potential target for aging intervention, remains largely uncharted in terms of its molecular functions within mammals. MRPs exhibit low amino acid similarity, making each MRP unique in function^52^. Through a series of experiments, it was determined that MRPL1 interacts with ATP5B and reduces its flexibility. This interaction inhibits ATP production and contributes to mitochondrial dysfunction. Mitochondrial dysfunction acts as a crucial determinant in the process of cellular senescence and serves as a prominent pathogenic factor in cartilage degeneration^15,16^. In particular, osteoarthritic chondrocytes exhibit impaired mitochondrial biogenesis and RC function^18,53^. The RC is responsible for energy metabolism through electron transfer facilitated by complexes I to IV, as well as ATP production via complex V (F_1_F_O_ ATP synthase)^19^. ATP synthase, a multisubunit enzyme complex comprising F_O_ (proton channel) and F_1_ (catalytic) domains, plays an essential role in ATP synthesis. This process accounts for the majority of cellular energy-consuming reactions and activities. ATP5B is the catalytic subunit of mitochondrial ATP synthases, and its dysfunction significantly hinders ATP synthesis, leading to increased mitochondrial protein turnover and senescence-related manifestations.

It is intriguing to note that the RC displays distinct expression patterns in chondrocytes under various pathophysiological conditions^17,54–58^. Dysfunction of complexes I–IV impairs electron transport during mitochondrial respiration, resulting in chondrocyte damage. For instance, mutations in mtDNA promote chondrocyte damage and cartilage degeneration^17,54,55^. Conversely, the inhibition of mitochondrial transcription suppresses mitochondrial respiration during cartilage development and protects chondrocytes from lethal intracellular anoxia^56^. Our findings indicate a notable resemblance to this, as both the administration of doxycycline and the knockdown of MRPL1 remarkably restrain mitochondrial respiration and hinder the senescence of chondrocytes. Mitochondrial ATP synthase serves as the primary executor of energy production, and its functionality possesses unparalleled significance. Inhibition of ATP synthase activity significantly aggravates chondrocyte dysfunction and amplifies inflammatory responses^57,58^. In the present study, the elevated expression of MRPL1 augments its interaction with ATP5B, which consequently suppresses ATP synthase activity. This ultimately results in an accelerated senescence of chondrocytes.

MRPL1, a mitoribosomal component involved in RC protein synthesis, is expected to enhance energy production when overexpressed. However, the results indicate that MRPL1 overexpression in EPCs paradoxically impairs the functionality of the RC, thereby serving as a mechanism of negative feedback. Correspondingly, we observed an increase in oxygen consumption following MRPL1 overexpression, indicating enhanced function of complexes I–IV. Nevertheless, ATP production decreased alongside diminished ATP synthase activity, lowered mitochondrial membrane potential, increased MPTP opening and atypical mitochondrial dynamics. The overall condition can be characterized by a state of mitochondrial uncoupling. It is noteworthy that this phenomenon has been suggested as a cytoprotective strategy under certain pathological conditions, particularly in the context of extreme oxidative stress, such as diabetes and cardiomyocyte injury^59,60^. Therefore, the specific roles of mitochondrial uncoupling in cartilage degeneration necessitate further investigation.

This study is subject to several additional limitations. On the one hand, the implications of in vivo gene editing of *Mrpl1* require additional experimental validation. On the other hand, MRPL1’s impact on other components of the RC remains to be explored. Furthermore, it is plausible that other MRP proteins may exert regulatory influences on ATP synthases, as indicated by our MS results. It is imperative that further explorations be undertaken to elucidate the specific molecular mechanisms that govern the dysregulation of RC within chondrocytes under diverse conditions.

**Fig. 8:**
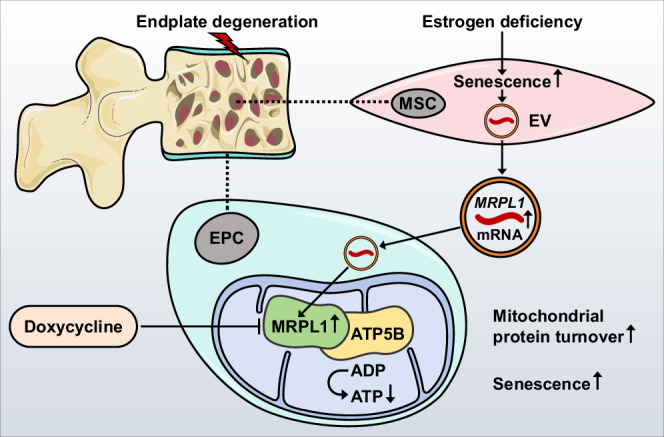
Schematic illustration depicting BMSC-EV-mediated pathological changes in endplate degeneration under estrogen deficiency. Vertebral BMSC-EVs deliver *MRPL1* mRNA to EPCs, promoting MRPL1 expression and inducing cellular senescence through enhanced mitochondrial protein turnover and inhibition of ATP synthase via interaction with ATP5B.

In conclusion, this study provides insights into the pathological effects of vertebral BMSC-EVs in inducing EPC senescence through the delivery of *MRPL1*, which restrains ATP5B function and can be reversed by doxycycline treatment. Our research establishes a connection between mitochondrial translation and endplate degeneration, thereby offering a new therapeutic strategy for the treatment of LDD (Fig. 8).

## Supplementary information

Supplementary Information
