# Supplementary material for: Vertebral BMSC-EVs under estrogen deficiency drive senescence-related mitochondrial dysfunction in endplate chondrocytes via MRPL1 mRNA delivery
Source: Exp Mol Med. 2026 May 7;58(5):1522–35. doi: 10.1038/s12276-026-01719-x (PMC13234282; doi:10.1038/s12276-026-01719-x)
Supplement: Supplementary file 1 — Supplementary Information [file 12276_2026_1719_MOESM1_ESM.pdf]

Supplementary Materials for

# Vertebral BMSC-EVs under estrogen deficiency drive senescence-related mitochondrial dysfunction in endplate chondrocytes via *MRPL1* mRNA delivery

Yiming Zhong et al.

Corresponding authors: Huijie Leng, lenghj@bjmu.edu.cn; Weishi Li, puh3liweishi@163.com

**This file includes:**

Supplementary figure 1 to 6

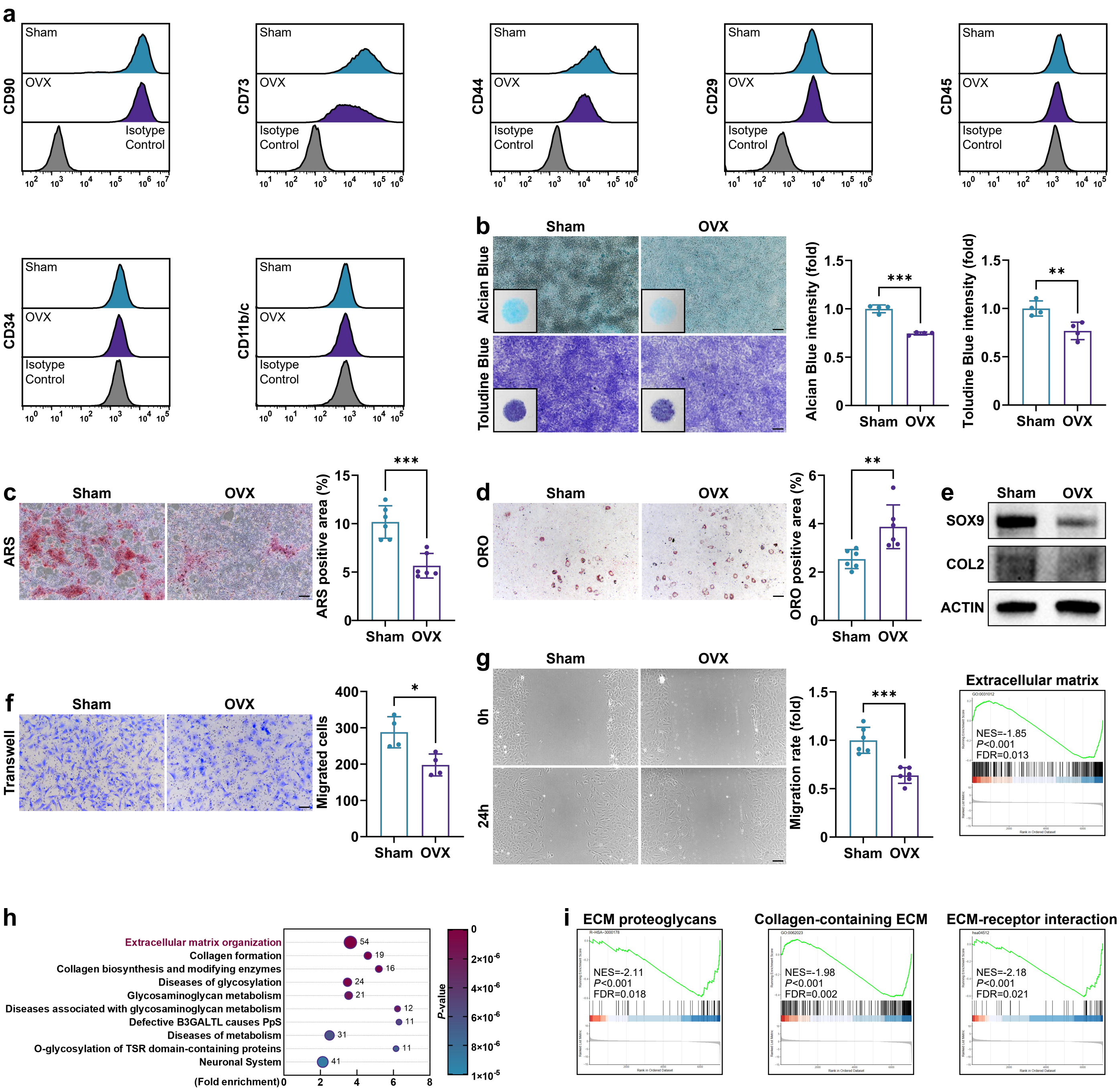


**Supplementary Fig. 1 Characterization of vertebral BMSCs.** **a** Flow cytometry analysis of the surface markers of BMSCs derived from vertebrae of sham-operated and ovariectomized (OVX) rats. **b** Alcian Blue and Toluidine Blue staining of BMSCs after chondrogenic differentiation ($n = 4$). **c** Alizarin Red S (ARS) staining of BMSCs after osteogenic differentiation ($n = 6$). **d** Oil Red O (ORO) staining of BMSCs after adipogenic differentiation ($n = 6$). **e** Western blot analysis of BMSCs after chondrogenic induction. **f** Transwell assay of BMSCs ($n = 4$). **g** Scratch assay of BMSCs ($n = 6$). **h** Reactome enrichment analysis of BMSCs from elderly women compared with age-matched men. **i** GSEA analysis of BMSCs from elderly women compared with age-matched men. Error bars represent mean ± SD. $^*P < 0.05$, $^{**}P < 0.01$, $^{***}P < 0.001$. Scale bar = 100 μm.

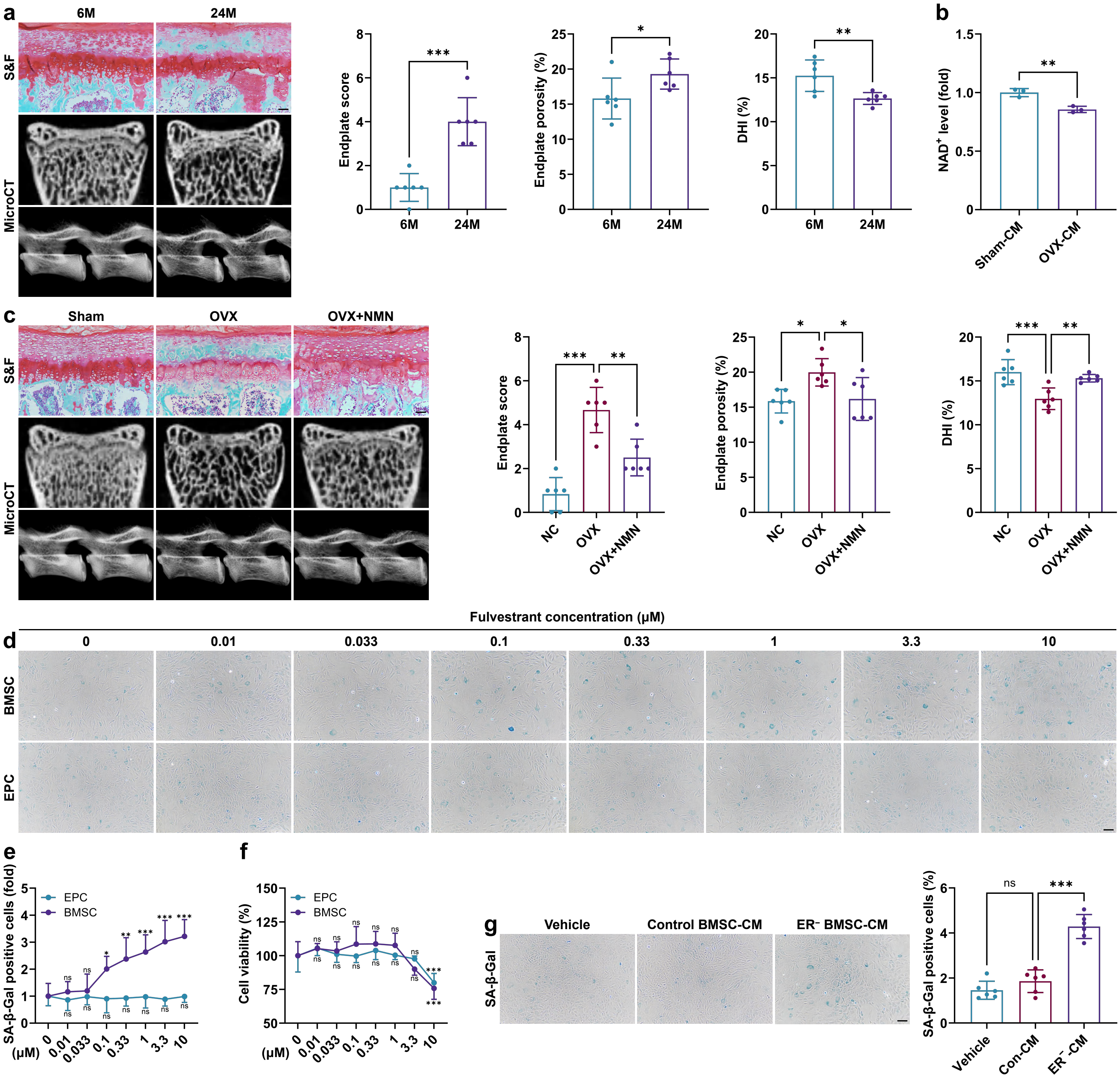


**Supplementary Fig. 2 Estrogen deficiency-induced BMSC senescence mediates endplate degeneration. a, c** Safranin O/Fast green (S&F) staining and microCT analysis of rat endplates ($n$ = 6). M: month. DHI: intervertebral disc height index. Scale bar = 50 µm. **b** $NAD^+$ levels of EPCs after BMSC conditioned medium (CM) treatment (3 d, $n$ = 3). **d, e** SA-β-Gal staining of BMSCs and EPCs after fulvestrant treatment (3 d, $n$ = 6)**.** Scale bar = 100 µm. **f** Cell viability of BMSCs and EPCs (3 d, $n$ = 6). **g** SA-β-Gal staining of EPCs after BMSC-CM treatment (3 d, $n$ = 6)**.** Scale bar = 100 µm. Error bars represent mean ± SD. *$P$ < 0.05, **$P$ < 0.01, ***$P$ < 0.001, ns: not significant.

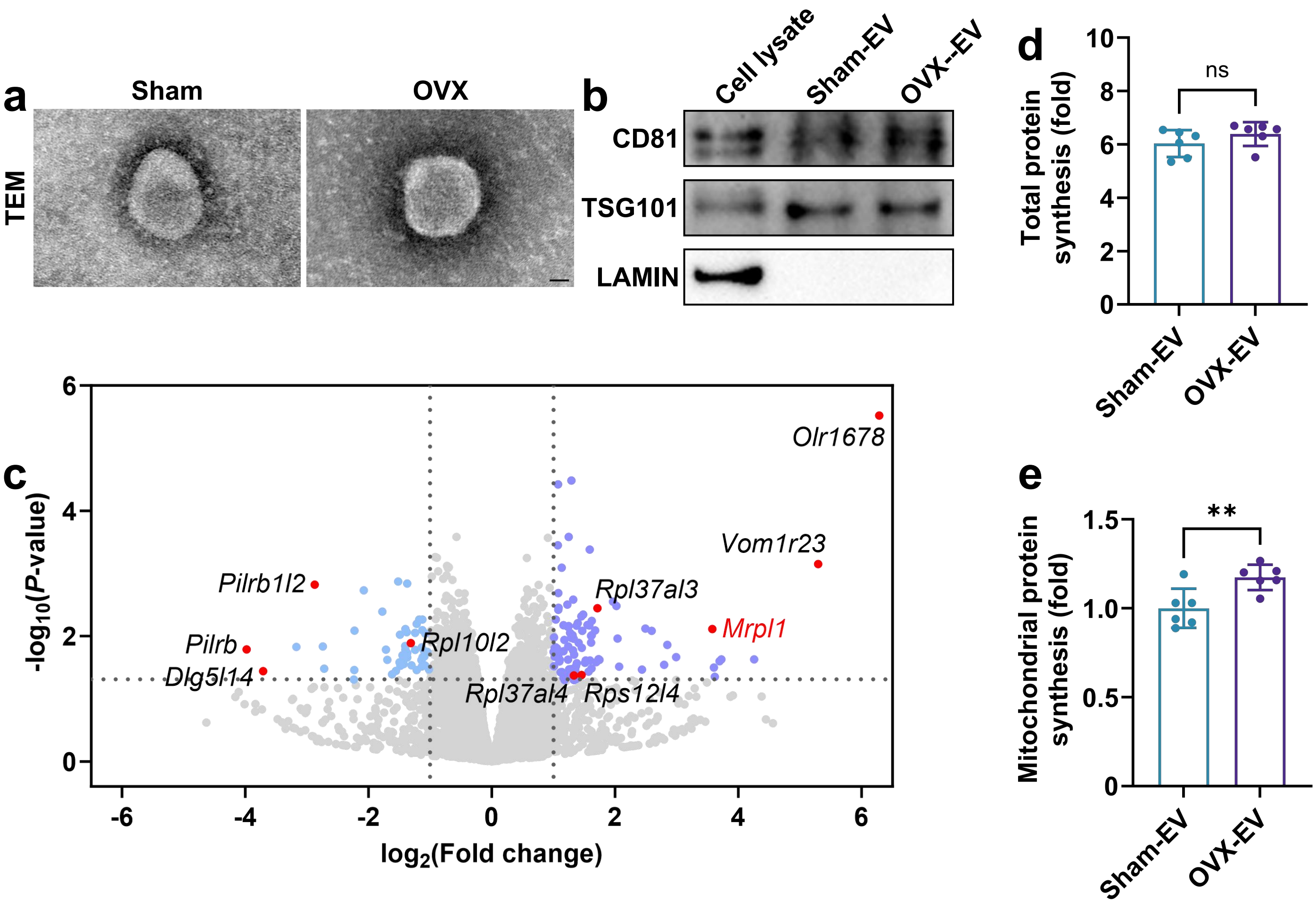


**Supplementary Fig. 3 Characterization and functional effects of vertebral BMSC-EVs. a** Transmission electron microscopy (TEM) of EVs from vertebral BMSC medium. **b** Western blot analysis of BMSC lysates and BMSC-EVs. **c** Volcano plot of differentially expressed mRNAs in OVX-EVs compared with Sham-EVs. **d** Total protein synthesis of EPCs after BMSC-EV treatment (3 d, $n = 6$). **e** Mitochondrial protein synthesis of EPCs after BMSC-EV treatment (3 d, $n = 6$). Error bars represent mean ± SD. $^{**}P < 0.01$, ns: not significant. Scale bar = 20 nm.

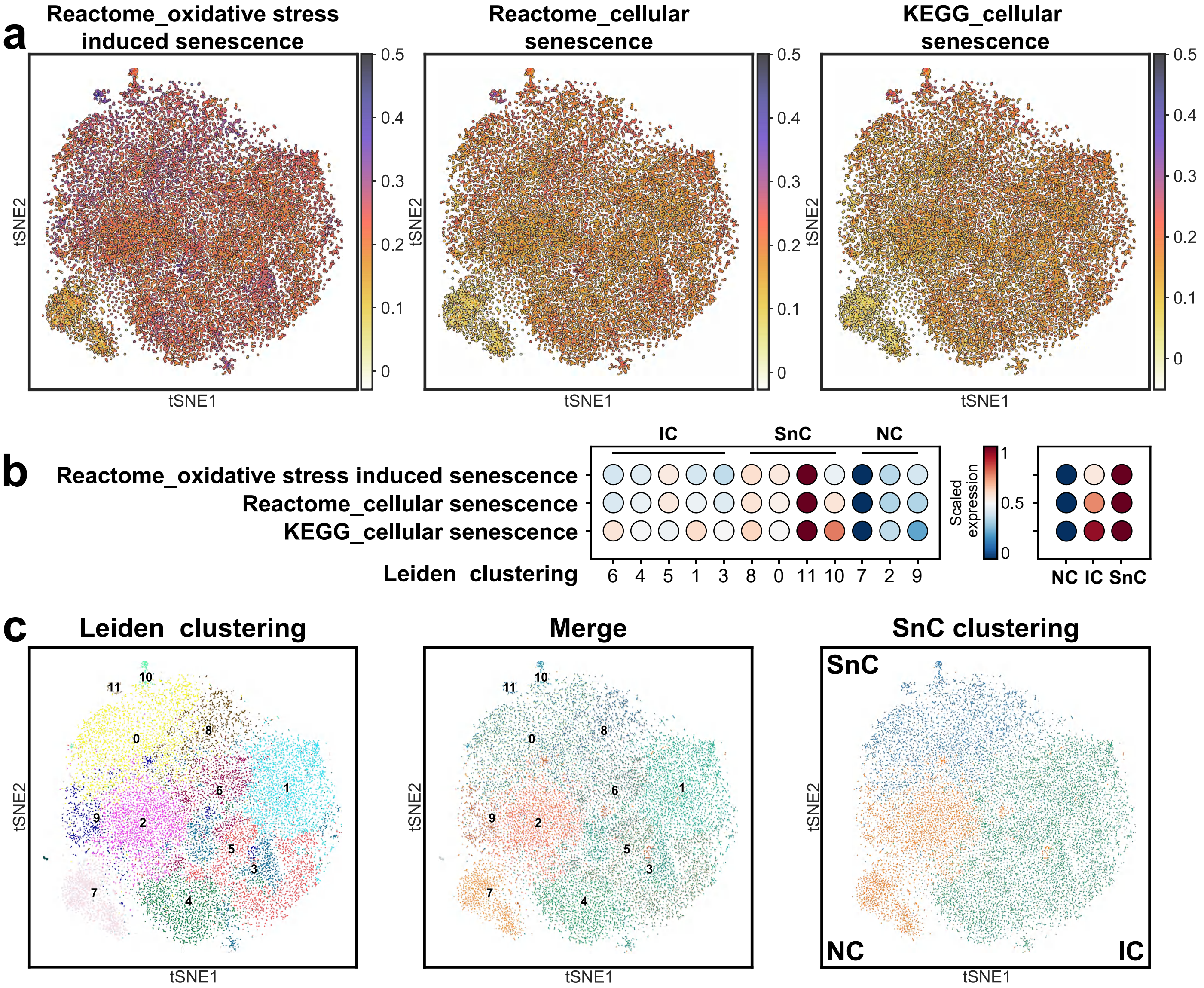


**Supplementary Fig. 4 Clustering of senescent EPCs. a, b** Scoring of senescence-related pathways. NC: normal chondrocytes. IC: intermediate chondrocytes. SnC: senescent chondrocytes. **c** The clustering of EPCs by senescence-related pathway results.

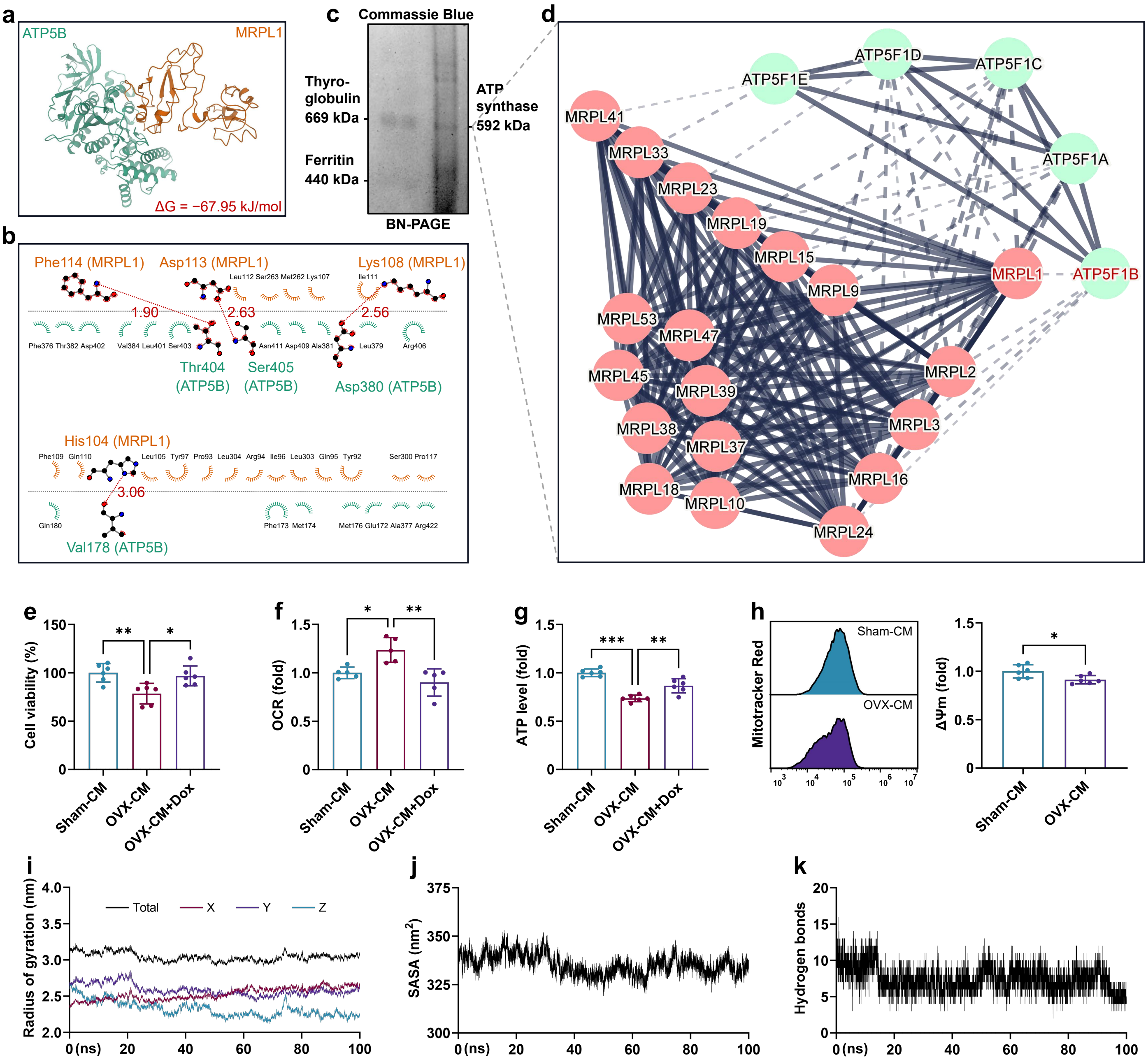


**Supplementary Fig. 5 Validation of MRPL1 and ATP5B interaction. a, b** Molecular docking of MRPL1 and ATP5B. **c** BN-PAGE separation of ATP synthases. **d** STRING analysis of mitoribosomal large subunit proteins and ATP synthase F1 subunit proteins detected by mass spectrometry in ATP synthase complexes separated by BN-PAGE. **e** Cell viability of EPCs after BMSC-CM and doxycycline treatment (3 d, $n = 6$). **f** Oxygen consumption rate (OCR) of EPCs (3 d, $n = 5$). **g** ATP levels of EPCs (3 d, $n = 6$). **h** Mitochondrial membrane potential of EPCs (3 d, $n = 6$). **i-k** Molecular dynamics simulation of the MRPL1-ATP5B complex. *$P < 0.05$, **$P < 0.01$, ***$P < 0.001$.

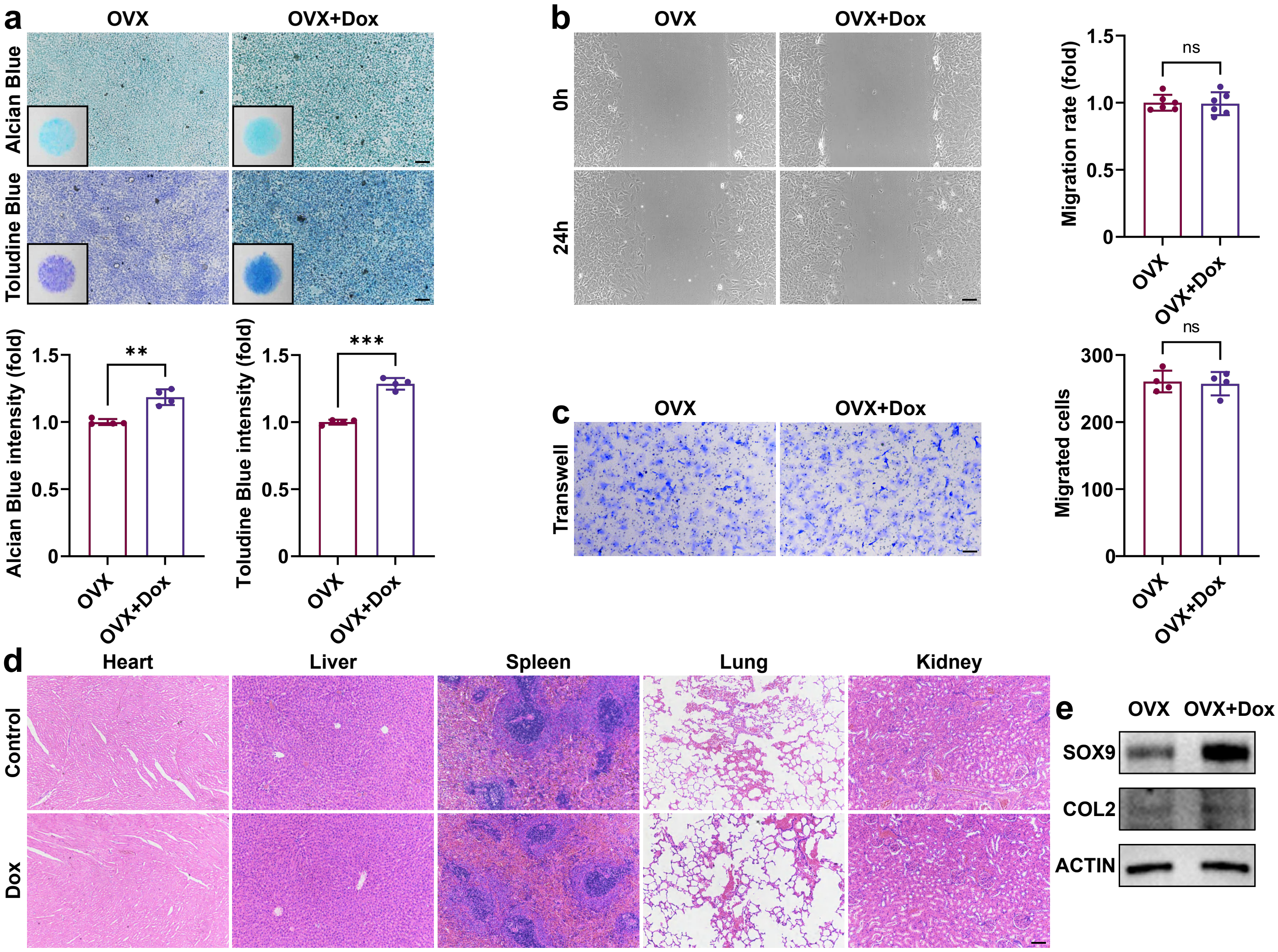


**Supplementary Fig. 6 Pharmacological effects of doxycycline. a** Alcian Blue and Toluidine Blue staining of BMSCs after chondrogenic differentiation ($n$ = 4). **b** Scratch assay of BMSCs ($n$ = 6). **c** Transwell assay of BMSCs ($n$ = 4). **d** Hematoxylin-Eosin (HE) staining of rat heart, liver, spleen, lung and kidney after doxycycline treatment. **e** Western blot analysis of BMSCs after chondrogenic differentiation. Error bars represent mean ± SD. $^{**}P < 0.01$, $^{***}P < 0.001$. ns: not significant. Scale bar = 100 μm
